# Update on the management of elderly patients with colorectal cancer

**DOI:** 10.1007/s12094-023-03243-0

**Published:** 2023-07-27

**Authors:** Gemma Soler-González, Javier Sastre-Valera, Antonio Viana-Alonso, Jorge Aparicio-Urtasun, Ignacio García-Escobar, María Auxiliadora Gómez-España, Carmen Guillén-Ponce, María José Molina-Garrido, Regina Gironés-Sarrió

**Affiliations:** 1 Departamento de Oncología Médica, Spanish Society of Medical Oncology (SEOM) Oncogeriatrics Section, Institut Català d’Oncologia (ICO) L’Hospitalet, Avinguda de la Granvia de l’Hospitalet 199-203, L’Hospitalet de Llobregat, 08908 Barcelona, Spain; 2 Spanish Cooperative Group for the Treatment of Digestive Tumours (TTD), Clinico San Carlos University Hospital, Madrid, Spain; 3 Spanish Society of Medical Oncology (SEOM) Oncogeriatrics Section, Nuestra Señora del Prado General University Hospital, Talavera de la Reina, Spain; 4 Multidisciplinary Spanish Group of Digestive Cancer (GEMCAD), Polytechnic la Fe University Hospital, Valencia, Spain; 5 Spanish Society of Medical Oncology (SEOM) Oncogeriatrics Section, General University Hospital of Toledo, Toledo, Spain; 6 Spanish Society of Medical Oncology (SEOM) Oncogeriatrics Section, Reina Sofía University Hospital. Instituto Maimónides de Investigación Biomédica de Córdoba (IMIBIC), Córdoba, Spain; 7 Spanish Society of Medical Oncology (SEOM) Oncogeriatrics Section, Ramón y Cajal University Hospital, Madrid, Spain; 8 Spanish Society of Medical Oncology (SEOM) Oncogeriatrics Section, Virgen de la Luz Hospital, Cuenca, Spain; 9 Spanish Society of Medical Oncology (SEOM) Oncogeriatrics Section, Polytechnic la Fe University Hospital, Valencia, Spain

**Keywords:** Aged, Antineoplastic, Capecitabine, Geriatric oncology, Fluoropyrimidines, Treatment outcome

## Abstract

Colorectal cancer (CRC) is one of the most common tumours worldwide, and 70% of CRC patients are over 65 years of age. However, the scientific evidence available for these patients is poor, as they are underrepresented in clinical trials. Therefore, a group of experts from the Oncogeriatrics Section of the Spanish Society of Medical Oncology (SEOM), the Spanish Cooperative Group for the Treatment of Digestive Tumours, (TTD) and the Multidisciplinary Spanish Group of Digestive Cancer (GEMCAD) have reviewed the scientific evidence available in older patients with CRC. This group of experts recommends a multidisciplinary approach and geriatric assessment (GA) before making a therapeutic decision because GA predicts the risk of toxicity and survival and helps to individualize treatment. In addition, elderly patients with localized CRC should undergo standard cancer resection, preferably laparoscopically. The indication for adjuvant chemotherapy (CT) should be considered based on the potential benefit, the risk of recurrence, the life expectancy and patient comorbidities. When the disease is metastatic, the possibility of radical treatment with surgery, radiofrequency (RF) or stereotactic body radiation therapy (SBRT) should be considered. The efficacy of palliative CT is similar to that seen in younger patients, but elderly patients are at increased risk of toxicity. Clinical trials should be conducted with the elderly population and include GAs and specific treatment plans.

## Introduction

Colorectal cancer (CRC) is one of the most common tumours worldwide [1, 2]. According to GLOBOCAN 2020 data, it is the third most prevalent tumour among both sexes after breast and lung neoplasia and the second leading cause of death from cancer after lung cancer [3]. Incidence and prevalence vary by country depending on lifestyles and screening programmes [2]. The risk of CRC increases with age; population ageing and overcoming other competing causes of death, such as cardiovascular diseases, are important factors [2]. The elderly population, therefore, is at high risk of developing CRC. This poses a challenge for healthcare organizations due to the presence of factors associated with ageing, such as frailty or comorbidities, which increase the risk of toxicity, impacting decision-making, especially as the algorithms for colon cancer treatment increase in complexity with age [2]. The heterogeneity of ageing generates discrepancies between chronological age and physiological age, making it essential to incorporate geriatric tools in decision-making for this population group.

To examine the management of the elderly population with CRC cancer in more detail, a group of experts from the Oncogeriatrics Section of the Spanish Society of Medical Oncology (SEOM), the Spanish Cooperative Group for the Treatment of Digestive Tumours (TTD) and the Multidisciplinary Spanish Group of Digestive Cancer (GEMCAD) have carried out an exhaustive review of the available scientific evidence, from which this publication arises.

## Categorization of elderly patients with CRC

Geriatric assessment (GA) is the basic work tool used in geriatrics. It allows a multidimensional evaluation, using different scales and questionnaires, of the health status of elderly patients. As a whole, a GA allows clinicians to identify the functional status of an individual, their state of mind, social and economic situation, and nutritional state, the presence of polypharmacy, and mobility, cognition and geriatric syndromes, among other aspects.

**Fig. 1:**
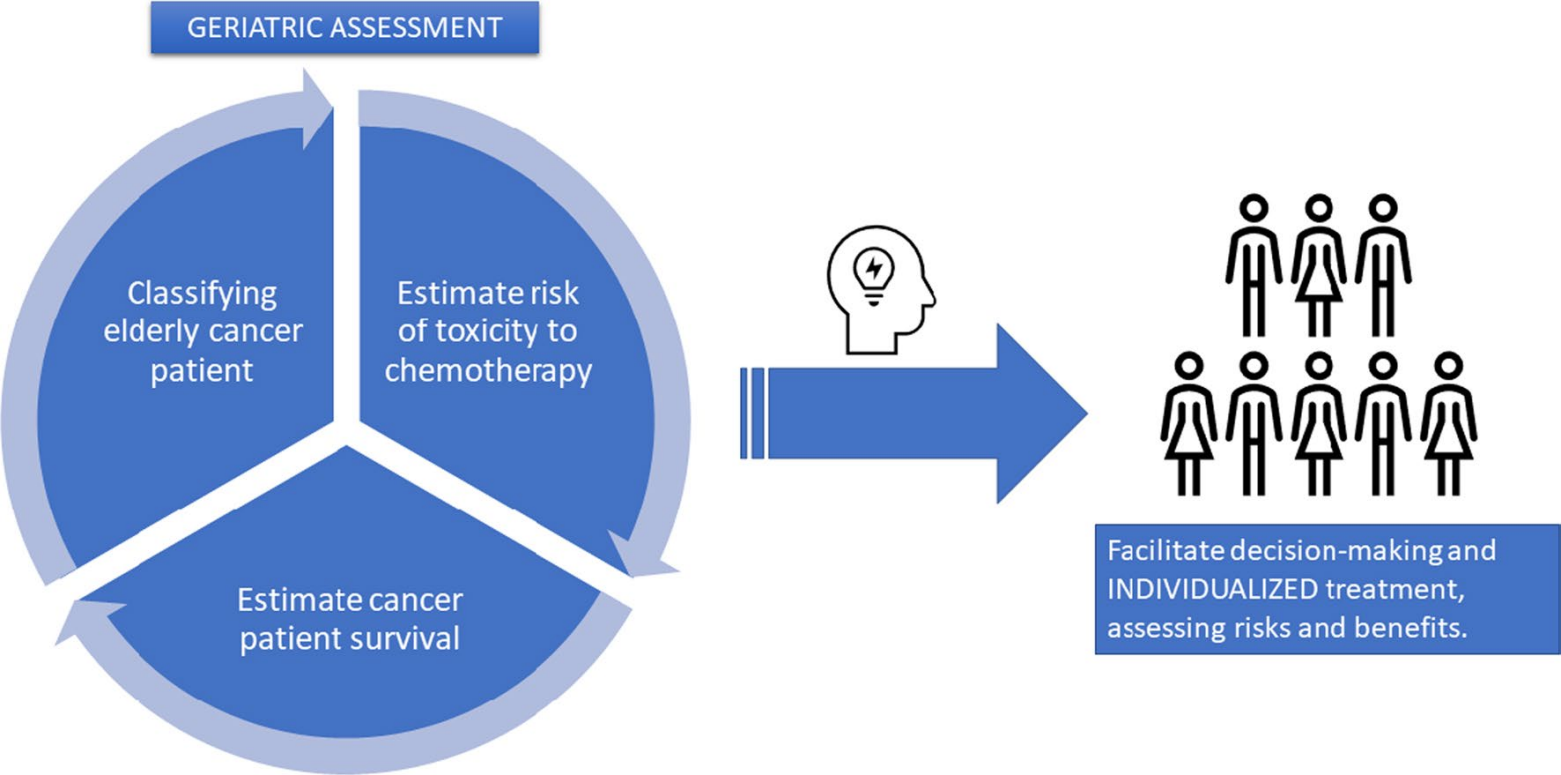
Categorization of elderly patients with CRC. *CRC* colorectal cancer

In the field of oncogeriatrics, the International Society of Geriatric Oncology (SIOG) recommends that a GA be performed for cancer patients over 70 years of age [4]. Through this GA, relevant information can be obtained for the development of a treatment plan that allows the most appropriate management for each patient (Fig. 1).

Since Professor Balducci’s proposal in 2000 to categorize elderly patients with cancer using data from the comprehensive geriatric assessment (CGA) [5], different classifications have been published, all of which have focused on identifying frail patients through the combination of different GA dimensions, such as functional capacity, nutritional status, comorbidities and geriatric syndromes [6]. All these classifications have emerged from consensuses among experts and from clinical experience. However, Ferrat et al*.* in 2016 used a statistical approach, i.e., latent class analysis (LCA), to identify frail patients from GA components [7].

In theory, frail patients tend to require more attention and care, as they are less able to cope with stressful situations, such as surgery or chemotherapy (CT). In fact, frail patients develop a higher incidence of post-surgical complications and a poorer tolerance to cytostatics [8]. For this reason, adapted treatment should be administered, and close follow-up should be conducted, together with the implementation of other intervention measures [9].

In the specific case of individuals with CRC, on whom this review focuses, the prevalence of frailty in the published series ranges from 20 to 43%; in all series, this population group has poorer survival and a greater number of severe complications after surgery [10, 11]. These results have also been described in a systematic review by Boakye et al*.* [12] and in a meta-analysis by Chen et al*.* [13], both in CRC.

**Fig. 2:**
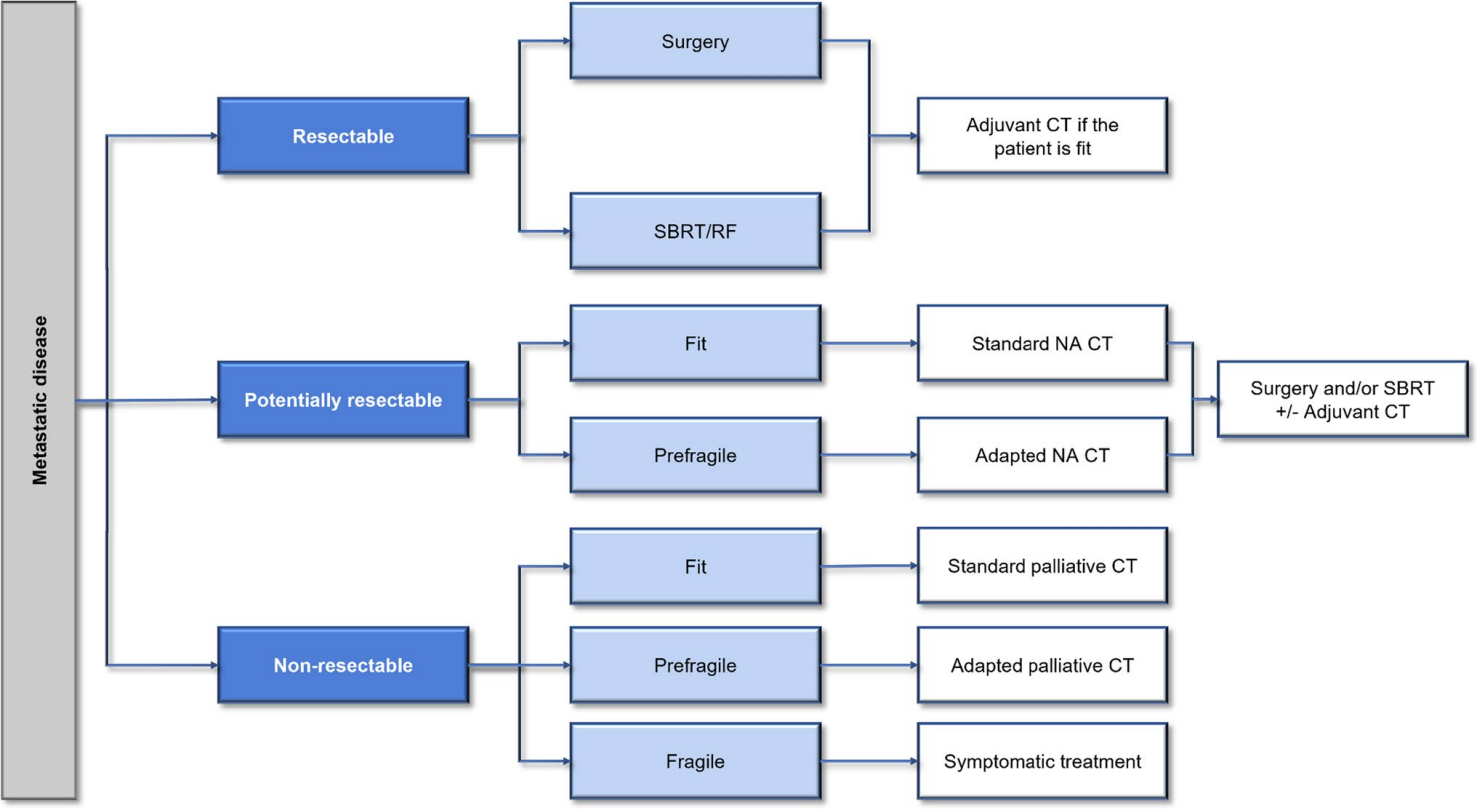
Decision algorithm for the treatment of elderly patients with metastatic CRC based on resectability. *CRC* colorectal cancer, *CT* chemotherapy, *NA* neoadjuvant, *RF* radiofrequency, *SBRT* stereotactic body radiation therapy

**Fig. 3:**
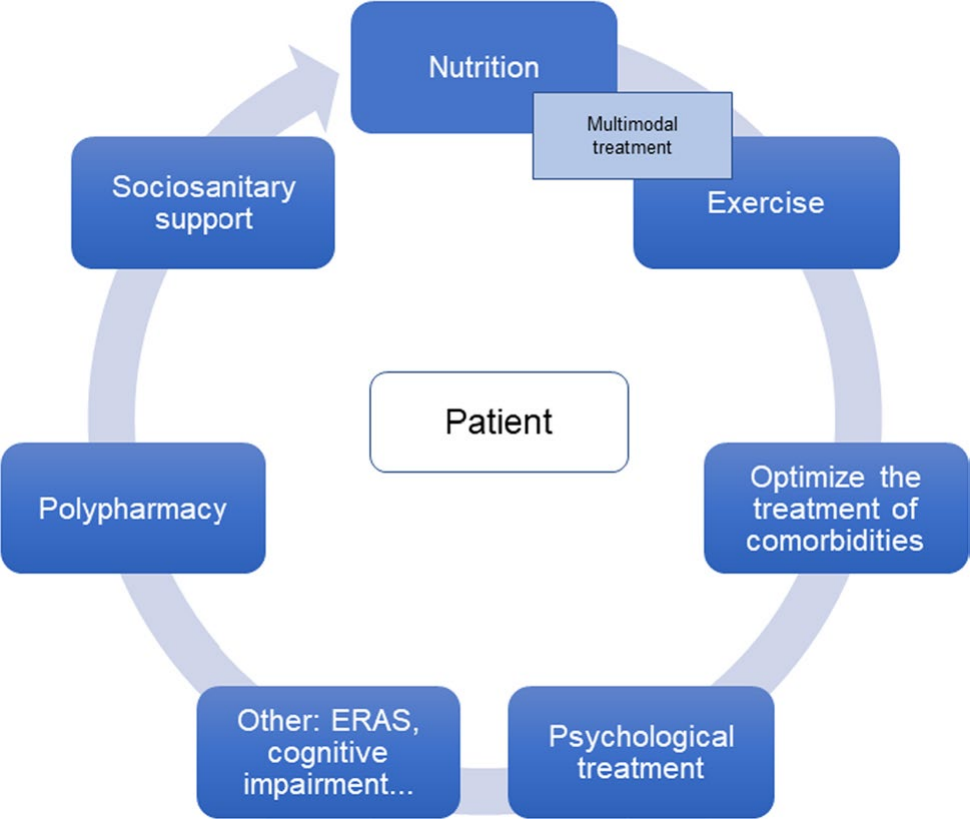
GA in elderly patients with CRC. *CRC* colorectal cancer, *ERAS* enhanced recovery after surgery, *GA* geriatric assessment

However, these classifications would be meaningless if they only served to catalogue patients. In contrast, antitumour treatment can be adapted depending on the presence or absence of frailty in patients with CRC, which favours tolerance to antitumour treatment, increases the probability that these patients will complete the treatment plan initially established, results in less frequent treatment dose reductions and improves patient quality of life [9, 14, 15]. Table 1 summarizes the main treatment options in elderly patients with localized CRC, taking into account their baseline situation.

**Table 1:** Therapeutic recommendations for elderly patients with localized CRC depending on their functional status

Patient	Colon cancer	Rectal cancer
Fit	1° Standard surgery 2° Adjuvant CT with fluoropyrimidines Alternative: XELOX for 3 months	1° 5 × 5 RT or CT with prolonged RT 2° Standard surgery Alternative: total neoadjuvant treatment
Pre-fragile	1° Standard surgery and perioperative rehabilitation 2° Adjuvant CT with fluoropyrimidines at reduced doses	1° Long course CT-RT and toxicity monitoring 2° Standard surgery and perioperative rehabilitation
Fragile	1° Palliative surgery 2° No adjuvant CT	1° RT 5 × 5 Alternative: derivative colostomy

*CRC* Colorectal cancer, *CT* chemotherapy, *RT* radiotherapy, *XELOX* capecitabine plus oxaliplatin

## Treatment of localized disease

Mortality from competing causes is higher among elderly patients than among younger patients with CRC. Therefore, although the time to recurrence is similar in both population groups, other efficacy variables, such as overall survival (OS) or disease-free survival (DFS), are not as favourable. A Swedish population study showed that the risk of recurrence after surgery, adjusted for the administration or not of adjuvant CT, was similar between elderly patients and the global population [16]. Therefore, the benefit of adjuvant CT after CRC surgery to reduce the risk of recurrence in the elderly is the same in elderly patients as in the rest of the population. In the absence of prospective randomized studies conducted specifically in this population, the only available scientific evidence comes from retrospective analyses performed with elderly patients with a good functional status that have been included in larger randomized clinical trials.

### Colon cancer

#### Surgery

Generally, elderly patients with CRC are assigned elective (scheduled) surgery less frequently than are younger patients; palliative intervention is more common. Although standard surgical procedures have proven to be tolerated in the elderly population, postoperative complications are more frequent, especially in frail patients. A comprehensive geriatric assessment (CGA) is therefore essential and should be incorporated into the preoperative assessment of geriatric patients with CRC.

Laparoscopic surgery has gained ground in recent years due to its lower postoperative morbidity, faster recovery of intestinal function and shorter hospital stay. This technique has also demonstrated its efficacy and safety in elderly patients with colon and rectal cancer [17]. Therefore, elderly patients with localized CRC and surgical indications and good functional status should undergo standard cancer resection, preferably laparoscopically. In cases with prefrailty, perioperative multimodal rehabilitation is recommended to reduce morbidity [18].

#### Adjuvant CT

Elderly patients with CRC are referred to Medical Oncology less frequently than are younger people, receive standard CT scans more rarely and are often given reduced doses or end treatment early.

In two joint analyses of 7 studies that evaluated adjuvant CT with fluoropyrimidines with that of surgery without CT in colon cancer, the benefits were similar between patients older than 70 years and the global population, without a significant increase in toxicity [19, 20]. Subsequently, a significant benefit has been demonstrated by adding oxaliplatin to the adjuvant regimens for CRC in these patients. In a pooled analysis of 4 randomized trials, DFS and OS improved significantly with the administration of oxaliplatin; however, in patients older than 70 years, this benefit was lower, and greater toxicity was observed [21]. One series of noninferiority trials, grouped under the umbrella of the International Duration Evaluation of Adjuvant Chemotherapy (IDEA), compared 3 and 6 months of adjuvant treatment in 12,834 patients with stage III colon cancer [22]. For various subgroups of patients (T3N1), 3 months of CT with capecitabine and oxaliplatin (XELOX) was not less effective than 6 months of the same treatment. The effect of age was not considered in any of these studies.

Therefore, the indication for adjuvant CT should be considered in the context of potential benefits, risk of recurrence, and life expectancy in relation to age and comorbidities. Elderly patients with resected stage III CRC (or stage II with poor prognosis) and good functional status should receive adjuvant CT with fluoropyrimidines for 6 months (or XELOX for 3 months). In prefrail patients, although there are no specific studies to support this approach, the administration of capecitabine at reduced doses with close monitoring for toxicity might be an option to improve tolerance. Frail patients are candidates for follow-up without complementary therapy. There are no published studies of adjuvant treatment in elderly patients; however, the French PRODIGE 34 ADAGE trial is under way.

### Rectal cancer

The management of localized rectal cancer is complex and requires a specialized multidisciplinary team. Standard treatment consists of long-term radiotherapy *(*RT) concomitant with fluoropyrimidines, followed by surgical resection (total excision of the mesorectum). This neoadjuvant treatment is associated with better local control and less toxicity [23]. The value of adjuvant CT after neoadjuvant CT and surgery is more questionable. Short-course RT (5 × 5) is a simpler alternative and does not require concomitant CT. However, it is not the best option in elderly patients who are candidates for surgery because it is associated with a higher rate of complications [24]. Recently, the value of total neoadjuvant treatment in conjunction with induction or preferably consolidation CT has been demonstrated, with the option of preserving the rectum provided there is close monitoring [25]. However, data for the elderly population are very limited.

In a review of the Swedish Rectal Cancer Registry, patients older than 75 years received preoperative RT and resection surgery less frequently. In addition, when this was carried out, the Hartmann technique (with definitive colostomy) was the approach that was used most frequently [26]. In general, after a preoperative assessment of faecal continence and a discussion of the potential risks of defecation urgency and faecal losses, the option of sphincter-conserving surgery should be offered to elderly patients with lower-third rectal cancer [27]. As the function of the anal sphincter may be lost with aging, sphincter-preserving surgery may not be considered in some cases. Importantly, all cases must be evaluated within a multidisciplinary team.

For nonfrail elderly patients, standard neoadjuvant treatment (long-course CT or short-course RT) is recommended, followed by conventional surgery (sphincter-sparing or abdominoperineal amputation, depending on baseline sphincter tone). Furthermore, in prefrailty situations, care should be taken to identify early signs of toxicity and adjust the treatment dose in a timely manner. In frail patients, the possible options are (i) discharge colostomy; (ii) palliative RT (5 × 5 technique); and (iii) symptomatic treatment.

## Treatment of advanced disease

Approximately 15–30% of CRC patients present with metastatic disease, and 20–40% of patients will metachronously develop metastases. The most frequent metastatic sites are the liver, lung, peritoneum, and distant lymphadenopathy. It is vitally important to assess and discuss the situation of each patient within a multidisciplinary committee of experts to establish the best individualized therapeutic option [28]. In addition, it is important to define whether the metastatic disease is resectable, potentially resectable after induction treatment, or unresectable; if unresectable, only palliative CT should be considered [29] (Fig. 2).

### First-line treatment with CT

Elderly patients with metastatic CRC have a lower OS than do younger patients because of multiple factors, including more advanced stage at diagnosis, more comorbidities and a greater frequency of suboptimal treatments [30, 31]. International guidelines, such as those released by the European Society for Medical Oncology (ESMO), prioritize establishing patient status during decision-making; in elderly patients, status is closely linked, although not exclusively, with comorbidities and functional reserves. In this sense, 3 subgroups of patients can be defined: fit, vulnerable or prefrail, and fragile [32].

#### Fit elderly patients

The ESMO guidelines recommend that chemotherapy treatment for fit elderly patients should be conducted in a similar way as that for younger patients, on the basis of results obtained in phase II studies and in age subgroup analyses in phase III trials, most of which include a fit elderly patient subgroup.

Some retrospective studies have evaluated 5-fluorouracil (5FU) and leucovorin (LV) with or without irinotecan (IRI) in elderly patients. Notably, a meta-analysis was conducted of 4 first-line treatment studies comparing 5FU/LV/IRI versus 5FU/LV in 2691 patients (599 ≥ 70 years). The same benefits in response rate and progression-free survival (PFS) were observed with the addition of IRI as in the control group, with a nonsignificant tendency towards higher OS in the elderly group. Toxicity was similar, except in patients older than 75 years, who had a higher incidence of severe neutropenia with monotherapy and a higher incidence of nausea, vomiting and diarrhoea in the IRI arm [33].

A subanalysis of the BICC-C study, which included 1,430 patients randomized to receive irinotecan with 5FU and folinic acid in continuous infusion (FOLFIRI), irinotecan with 5FU-bolus and folinic acid (mIFL) or irinotecan and capecitabine (CapeIRI), showed that the response and PFS for patients older than 70 years were similar to those for younger patients, although with a higher incidence of asthenia and dehydration related mainly to capecitabine [34]. Other prospective studies in which irinotecan regimens have been evaluated have confirmed their feasibility and efficacy [35].

In analyses by population subgroup, the efficacy/safety relationship has been shown to remain positive in fit elderly patients treated with oxaliplatin-based regimens, as demonstrated by a meta-analysis of 3742 patients (614 ≥ 70 years) in 4 phase III studies that evaluated the combination of folinic acid, 5FU and oxaliplatin (FOLFOX) as first- and second-line adjuvant treatment for advanced disease, detecting no differences in terms of efficacy or toxicity (except in some haematological parameters) with respect to the global population of the study [36]. A subanalysis of the phase III study 03/TTD/01, which compared 5FU and oxaliplatin (FUOX) with XELOX in 348 patients (109 ≥ 70 years), also did not show differences between the elderly population and the younger population in terms of efficacy or toxicity [37]. Prospective studies carried out in the elderly population confirm that the activities of the FOLFOX or XELOX combinations in elderly patients are similar to those in the younger population [38–40].

Two randomized phase III studies of elderly populations evaluated whether combined treatment provides any benefit compared with the administration of fluoropyrimidines as monotherapy. The FOCUS2 study recruited 459 patients not eligible for full-dose CT [41]. With a 2 × 2 factorial design, it included two arms with fluoropyrimidines in monotherapy and two arms with fluoropyrimidines in combination with oxaliplatin at reduced initial doses. The addition of oxaliplatin increased the response rate without an increase in PFS (5.8 vs. 4.5 months; *p* = 0.07) or OS. Substitution of 5FU for capecitabine did not improve patient quality of life. The addition of oxaliplatin did not significantly increase the incidence of grade 3/4 toxicity (38% vs. 32%; *p* = 0.17), which was observed when capecitabine was administered instead of 5FU (40% vs. 30%; *p* = 0.03). The FFCD 2001-02 trial compared FOLFIRI versus 5FU/LV administered in a classic or simplified way, without observing a benefit in terms of PFS or OS; however, greater toxicity was observed in the arms with irinotecan [42].

Therefore, clinicians need to carefully assess the results of GA in patients with metastatic CRC. Although the guidelines recommend administering similar treatments for nonelderly patients and fit elderly patients, in the presence of toxicity or a negative impact on quality of life, there is no clear evidence that polychemotherapy provides a clear survival rate benefit when compared to monotherapy.

#### Prefrail or vulnerable elderly patients

The benefit of administering fluoropyrimidines rather than supportive treatment has been clearly demonstrated in past decades [43]. Capecitabine offers an activity greater than 5FU-bolus and has a similar toxicity profile to 5FU administered as a continuous infusion, although with a better quality of life. A phase II study of patients ≥ 70 years of age treated with capecitabine as monotherapy reported a response rate of 24%, PFS of 7 months and OS of 11 months. There were no significant differences in the incidence of grade 3–4 toxicities between the general study population and patients older than 80 years [44].

Several phase II trials with modified treatment or dosage regimens have evaluated the administration of fluoropyrimidine-based polychemotherapy in this subgroup of patients. Low-dose FOLFOX regimens resulted in response rates of 43–51% and OS of 13–20 months [45, 46]. The administration of oxaliplatin at a reduced dose in the first cycle (85 mg/m^2^) and higher doses in the second cycle (100 mg/m^2^) together with capecitabine at standard doses resulted in toxicity and escalation in 51% of elderly patients with metastatic CRC at the initial assessment, with a response rate of 40% and an OS of 14 months [47].

The results from the NORDIC-9 randomized phase II trial suggest that, compared with a sequence of monotherapy with full doses, the sequence of first and second lines of polychemotherapy at a reduced dose leads to an increase in PFS, less toxicity and improved quality of life in the vulnerable elderly [48, 49].

#### Frail elderly patients

Current treatment guidelines advise against the use of CT in frail elderly patients with metastatic CRC [33].

### First-line treatment with CT and targeted therapy

The most relevant studies for this topic are shown in Table 2.

**Table 2:** Evaluation of first-line treatments in the elderly patient with metastatic CRC

Study	*N* Age	Treatment scheme	GA	Efficacy
Phase III AVEX [50]	N = 280 ≥ 70	A: Capecitabine B: Capecitabine + bevacizumab	No	PFS: 5.2 vs. 9.1 m; *p* < 0.0001 OS: 20.7 vs. 16.8 m; *p* = 0.18 RR: 19% vs. 10%; *p* = 0.04
Phase III SOLSTICE [51, 52]	N = 856 435 ≥ 75 (Fragile)	A: TAS-102 + bevacizumab B: Capecitabine + bevacizumab	Yes	PFS: 9.4 vs. 9.3 m; *p* = 0.0464 (superiority *p* < 0.021) OS: Pending RR: 36% vs. 42%; *p* = 0.0942
Phase III [53] JCOG1018	N = 251 ≥ 70	A: FP + bevacizumab B: FP + oxaliplatin + bevacizumab	Yes	PFS: 9.4 vs. 10 m; *p* = 0.086 OS: 21.3 vs. 19.7 m RR: 30% vs. 48%
Phase II-III AGITG MAX [54]	N = 99 Subgroup ≥ 75	C: Capecitabine CB: Capecitabine + bevacizumab CBM: Capecitabine + bevacizumab + Mitomycin C	No	PFS CB vs. C: 0.52 (0.32–0.86); *p* = 0.01 PFS CBM vs. C: 0.38 (0.23–0.64); *p* = 0.001 OS CB vs. C: 0.80 (0.47–1.36); *p* = 0.41 OS CBM vs. C: 0.78 (0.46–1.34); *p* = 0.36 RR C vs. CB vs. CBM: 28% vs. 23% vs. 57%; *p* = 0.08
Phase II [55] PRODIGE 20	N = 102 ≥ 75	A: FOLFOX/FOLFIRI/ 5FU-LV B: FOLFOX/FOLFIRI/5FU-LV + bevacizumab	No	PFS: 7.8 vs. 9.7 m; HR 0.79 [95% Cl 0.53–1.17] OS: 19.8 vs. 21.7 m; HR = 0.73 [95% Cl 0.48–1.11] RR: 33% vs. 37%
Phase III-II AVF2107-AVF2192 pooled [56]	N = 439 ≥ 65	A: 5FU-LV bolus B: 5FU-LV bolus + bevacizumab	No	PFS: 6.2 vs. 9.2 m; *p* < 0.001 OS: 14.3 vs. 19.3 m; *p* = 0.006 RR: 29% vs. 34%; *p* = ns
Phase II Sastre et al*.* [57]	N = 66 ≥ 70	Capecitabine + cetuximab	No	PFS: 8.4 RAS WT vs. 6 RAS mut; *p* = 0.024 OS: 8.4 RAS WT vs. 6 RAS mut; *p* = 0.024 RR: 48% RAS WT vs. 21% RAS mut; *p* = 0.027
Phase II PANDA [58]	N = 185 ≥ 70	A: FOLFOX + panitumumab B: 5FU/LV + panitumumab	Yes	PFS: 9.6 vs. 9.1 m OS: Pending RR: 65% vs. 57%; *p* = ns

*5FU* 5-fluorouracil, *CRC* colorectal cancer, *FOLFIRI* 5FU and folinic acid with irinotecan, *FOLFOX* 5FU and folinic acid with oxaliplatin, *FP* fluoropirimidina (5FU/capecitabina, *GA* geriatric assessment, *LV* leucovorin, *m* month, *mut* mutant, *OS* overall survival, *PFS* progression-free survival, *RAS* rat sarcoma virus, *TAS-102* trifluridine/tipiracil, *WT* wild-type, *RR* response rate

#### CT plus antiangiogenic treatment

The phase III AVEX study evaluated the administration of capecitabine with or without bevacizumab in 280 patients with metastatic CRC ≥ 70 years of age who were not candidates to receive oxaliplatin- or irinotecan-based regimens. The average age of the patients was 76 years. The median PFS was higher in the arm treated with capecitabine and bevacizumab than in the arm treated with capecitabine alone (9.1 vs. 5.2 months; *p* < 0.0001), with a trend towards higher OS (20.7 vs. 16.8 months; *p* = 0.18) and a higher response rate (19% vs. 10%; *p* = 0.04). The benefit was seen in both older and younger patients. The combination also caused higher grade 3–4 toxicity (40% vs. 22%) [50].

In vulnerable patients, the phase III SOLSTICE study compared the administration of bevacizumab and capecitabine with bevacizumab and trifluridine/tipiracil (TAS-102). However, no benefit was observed in the experimental arm; the PFS was 9.4 months versus 9.3 months, with a different toxicity profile (more neutropenia with TAS-102 and more hand-foot syndrome with capecitabine). The G8 scale was included for patients ≥ 70 years of age, and a trend towards greater toxicity was observed in patients with a score < 14 in the TAS-102 arm [51, 52].

The benefit of adding oxaliplatin to fluoropyrimidine and bevacizumab treatment for patients ≥ 70 years of age with metastatic CRC was evaluated in the phase III JCOG1018 (RESPECT) study, which included 251 patients. In the oxaliplatin arm, the PFS was 9.4 (10.0 months in the other arm; *p* = 0.086), the OS was 21.3 (19.7 months in the other arm), and the response rate was 29.5% (47.7% in the other arm), without differences in the quality of life of patients in the two arms. The authors did not recommend the addition of oxaliplatin to the combination of fluoropyrimidines and bevacizumab as a first-line treatment [53].

#### CT plus anti-EGFR treatment

Combinations of CT with epidermal growth factor receptor inhibitors *(*anti-*EGFR*), such as cetuximab and panitumumab, are less studied than are combinations with bevacizumab in elderly patients.

Papamichael et al*.* evaluated, from 7 clinical trials, the safety and efficacy of adding anti-*EGFR* treatment to first-line CT in 1920 patients (≥ 70 and < 70 years) with metastatic CRC without a *RAS* gene mutation. Older patients who received CT plus anti-*EGFR* had a PFS similar to younger patients (8.7 vs. 10.3 months; *p* = 0.107) with a lower OS (21.3 vs. 26.3 months; *p* = 0.011) and without significant differences regarding the incidence of toxicity. In the group of patients ≥ 70 years of age, when comparing CT plus anti-*EGFR* versus CT alone, no significant differences were observed in terms of PFS and OS. This may be due to the poorer prognosis of patients ≥ 70 years of age, with a higher percentage of patients with an Eastern Cooperative Oncology Group (ECOG) score > 1, a higher incidence of primary tumours located in the right colon, and a higher presence of pulmonary metastases [59].

A phase II study with 66 patients ≥ 70 years of age with metastatic CRC evaluated the combination of cetuximab and capecitabine. The response rate was 48.3% in patients without a *KRAS* mutation. The PFS was 8.4 months and the OS was 18.8 months. After the first 27 patients were included, a dose reduction on capecitabine was implemented due to toxicity [57].

In the retrospective REVOLT study, 118 vulnerable patients ≥ 70 years of age with metastatic CRC without *RAS* or *BRAF* mutations *(*B-Raf proto-oncogene*, BRAF*) who received FOLFOX or FOLFIRI at a reduced dose plus an anti-*EGFR* (cetuximab or panitumumab) were evaluated. The G8 score was ≤ 14 for all patients, the response rate was 57.3%, the PFS was 10.0 months, and the OS was 18.0 months. The most frequent grade 3–4 toxicities were neutropenia (11.8%) and skin toxicity (11%). These results support the administration of reduced-dose treatments to vulnerable populations [60].

The randomized phase II PANDA study compared the administration of panitumumab and FOLFOX with panitumumab and 5FU/LV (eliminating the 5FU bolus in both arms), followed by maintenance treatment with panitumumab in patients ≥ 70 years without *RAS* or *BRAF* mutations. G8 and CRASH were applied. The PFS was 9.6 versus 9.1 months in the FOLFOX and 5FU/LV arms, respectively, and the response rate was 65% versus 57% in the FOLFOX and 5FU/LV arms, respectively. A higher incidence of grade 3–4 toxicity was observed in the oxaliplatin arm (neutropenia, diarrhoea, and neurotoxicity). The OS results have not yet been communicated. The authors recommended administering the panitumumab with 5FU/LV regimen followed by a maintenance treatment with panitumumab as the best option for these patients until the results of the phase III studies are reported [58].

### Second-line or successive treatments with CT

There are no second-line or successive studies aimed at the elderly population; therefore, data are taken from analyses of subgroups by age, from which it is extracted that fit patients can receive the same CT regimen as younger patients (Table 3). The regimen should be chosen based on the first-line treatment administered. However, in prefrail and frail patients, monotherapy should be administered to reduce toxicity [61]. Second-line irinotecan in patients ≥ 70 years has shown efficacy and toxicity similar to that in younger patients [62].

The retrospective STREAM study included 873 patients with metastatic CRC between November 2016 and April 2017 in 33 Spanish hospitals. The treatment regimens most frequently received by patients > 75 years (15.2% of the total) were second line: (i) irinotecan (13%); (ii) capecitabine (10%); and (iii) FOLFIRI (10%). As third-line treatments, they received (i) FOLFOX (18%); (ii) FOLFIRI (8%); (iii) irinotecan plus cetuximab (8%); (iv) capecitabine (8%); and (v) panitumumab (8%). Fourth-line treatments included (i) capecitabine (32%); (ii) FOLFOX (16%); and (iii) regorafenib (16%) [63].

TAS-102 is an oral fluoropyrimidine indicated in patients previously treated with CT based on fluoropyrimidines, oxaliplatin and irinotecan, vascular endothelial growth factor inhibitors* (*anti-*VEGF*) and, in patients without *KRAS* mutations, with anti*-EGFR* treatments. In a subgroup analysis of a *post* hoc phase III study, the benefits of this therapy in elderly patients were confirmed. In addition, in other analyses carried out in real clinical practice, there were no differences either in efficacy or in the safety profile as a function of patient age [64, 65].

### Second-line or successive treatments with CT and targeted therapy

In a review reported by Altshuler E et al*.* it was observed that tumours of younger patients were less likely to have a microsatellite instability-high/deficient mismatch repair (MSI-H/dMMR) than tumours of older patients (12.5% vs. 21.4%, *p* = 0.013), similarly to *BRAF* mutation (1.5% vs. 16%, *p* = 0.002). *BRAF* mutation status was highly associated with MMR status. Thus, *BRAF* mutated tumours were 29.7 times more likely than B-RAF-WT tumours to be MSI-H/dMMR (*p* < 0.001) [66].

Patients with native *RAS* and *BRAF* CRC who have not received anti-*EGFR* therapy (cetuximab or panitumumab) as a first-line treatment can receive it alone or in combination with CT in successive lines, with acceptable tolerance in the elderly [67]. Evaluation of the *RAS* mutation status by analysing circulating tumour DNA (ctDNA) may be useful in identifying patients who are candidates for retreatment with anti-*EGFR* drugs in successive lines [68, 69] (Table 3).

**Table 3:** Evaluation of second-line and successive treatments for elderly patients with metastatic CRC

Study *N*	Treatment	Age, median (range)	Efficacy	Toxicity
Second-line treatment				
Phase III [62] Chau I et al. *N* = 339	Irinotecan monotherapy	62 (29–80)	All: OS: 9.1 m; < 70 vs. ≥ 70: no differences; *p* = 0.74 < 70: RR: 9.0%; 95% CI 5.6–12.4 ≥ 70: RR: 11.1%: 95% CI 4.9–20.7; *p* = 0.585	No differences < 70 vs. ≥ 70: 38% vs. 46%; *p* = 0.218
		< 70 yr: 79%		
		≥ 70 yr: 21%		
Phase III [70, 71] VELOUR *N* = 1226	Ratio 1:1 FOLFIRI + A or B A: Aflibercept B: Placebo	61 (19–86)	A vs. B OS: 13.50 vs. 12.06 m; HR: 0.82; *p* = 0.0032 OS < 65: HR: 0.80; 95% CI 0.67–0.95 OS ≥ 65: HR: 0.85; 95% CI 0.68–1.07 PFS: 6.90 vs. 4.67 m; *p* < 0.0001 PFS < 65: HR: 0.77; 95% CI 0.55–1.08 PFS ≥ 65: HR: 0.75; 95% CI 0.48–1.17 RR: 19.8% vs. 11.1%; *p* = 0.29	A vs. B ≥ G3: 84% vs. 63%
		< 65 yr: 783 (64%)		
		≥ 65 yr: 443 (36%)		
Phase III [72] RAISE *N* = 1072	Ratio 1:1 FOLFIRI + A or B A: Ramucirumab B: Placebo	62 (21–87)	A vs. B OS: 13.3 vs. 11.7 m; HR: 0.84; *p* = 0.0219 OS < 65: HR: 0.86; 95% CI 0.72–1.03 OS ≥ 65: HR: 0.85; 95% CI 0.68–1.07 PFS: 5.7 vs. 4.5 m; *p* < 0.0005 PFS < 65: HR: 0.82 95% CI 0.67–1.00 PFS ≥ 65: HR: 0.78 95% CI 0.67–0.92 RR: 13.4% vs. 12.5%; *p* = 0.63	A vs. B G3: 79% vs. 62%
		< 65 yr: 645 (60%)		
		≥ 65 yr: 427 (40%)		
Second-line and third-line treatment				
Phase III [73] RECOURSE *N* = 800	Ratio 2:1 A: TAS-102 B: Placebo	63 (27–82)	A vs. B OS: 7.1 vs. 5.3 m; HR: 0.68; *p* < 0.001 OS < 65: HR: 0.74; 95% CI 0.59–0.94 OS ≥ 65: HR: 0.62; 95% CI 0.48–0.80 PFS: 2.0 vs. 1.7 m; HR: 0.48; *p* < 0.001 PFS < 65: HR: 0.52; 95% CI 0.42–0.65 PFS ≥ 65: HR: 0.41; 95% CI 0.32–0.52 RR: 1.6% vs. 0.4%; *p* = 0.29	A vs. B ≥ G3: 69% vs. 52%
		< 65 yr: 448 (56%)		
		≥ 65 yr: 352 (44%)		
Phase III [74] CORRECT *N* = 760	Ratio 2:1 A: Regorafenib B: Placebo	61 (54–68)	A vs. B OS: 6.4 vs. 5.0 m; HR: 0,77; *p* = 0.0052 OS < 65: HR: 0.72; 95% CI 0.56–0.91 OS ≥ 65: HR: 0.86; 95% CI 0.61–1.19 PFS: 1.9 vs. 1.7 m; *p* < 0.0001 PFS < 65: HR: 0.42; 95% CI 0.34–0.51 PFS ≥ 65: HR: 0.65; 95% CI 0.50–0.86 RR: 1.0% vs. 0.4%; *p* = 0.19	A vs. B ≥ G3: 54% vs. 14%
		< 65 yr: 475 (63%)		
		≥ 65 yr: 285 (37%)		
Phase III [75] BEACON *N* = 665	Ratio 1:1:1 Cetuximab + A/B/C A: Encorafenib + Binimetinib B: Encorafenib C: Irinotecan or FOLFIRI	61 (26–91)	A vs. C OS: 9.3 vs. 5.9 m; HR: 0.60 OS < 65: HR: 0.61; 95% CI 0.45–0.81 OS ≥ 65: HR: 0.56; 95% CI 0.38–0.82 PFS: 4.5 vs. 1.5 m; HR: 0.42; 95% CI 0.33–0.53 RR: 26.8% vs. 1.8%; *p* < 0.0001 B vs. C OS: 9.3 vs. 5.9 m; HR: 0.61 OS < 65: HR: 0.56; 95% CI 0.42–0.76 OS ≥ 65: HR: 0.65; 95% CI 0.44–0.95 PFS: 4.3 vs. 1.5 m; HR: 0.44; 95% CI 0.35–0.55 RR: 19.5% vs. 1.8%; *p* < 0.0001	G ≥ 3 A vs. B vs. C: 66% vs. 57% vs. 64%
		< 65 yr: 290 (65%)		
		≥ 65 yr: 155 (35%)		

*CI* Confidence interval, *CRC* colorectal cancer, *FOLFIRI* irinotecan with 5-fluorouracil and folinic acid, *G* grade, *HR* hazard ratio, *m* month, *OS* overall survival, *PFS* progression free survival, *RR* response rate. *TAS-102* trifluridine/tipiracil, *yr* year

As a second-line antiangiogenic treatment, CT plus bevacizumab is feasible, even in patients who have previously progressed on bevacizumab and with native *RAS* tumours [76]. In the VELOR trial, which randomized patients to receive FOLFIRI with or without aflibercept as a second-line treatment after progression on an oxaliplatin regimen, 34% of patients ≥ 65 years were included in the combination arm with aflibercept, among whom 5% were ≥ 75 years. A survival benefit was observed with the combination of FOLFIRI plus aflibercept in patients ≥ 65 years of age, although with a higher incidence of diarrhoea, dehydration, asthenia and weight loss and permanent discontinued treatment due to adverse events [70]. However, studies carried out in real clinical practice showed contradictory data regarding the safety profile in elderly patients [77, 78].

The phase III RAISE trial, which administered FOLFIRI with or without ramucirumab after progression on first-line CT with bevacizumab, included 427 patients (40%) ≥ 65 years of age. In the subgroup analysis, the survival benefit was similar regardless of age [72]. In the phase III CORRECT trial, compared to placebo, the administration of the multikinase inhibitor regorafenib led to an increase in survival in patients previously treated with CT based on fluoropyrimidines, oxaliplatin, irinotecan, anti-VEGF and, in native *KRAS* patients, with anti-EGFR treatment. Subgroup analysis suggested a lower benefit of regorafenib in patients > 65 years of age [74]. In the single-arm phase IIIb CONSIGN trial, no differences in efficacy and toxicity were observed as a function of age. However, other studies have suggested less benefit in patients > 65 years of age with a worse safety profile [79, 80]. The results of the phase III SUNLIGHT study have recently been published [81]. In 492 patients with metastatic CRC treated with 1–2 lines of previous CT, a survival benefit was demonstrated for patients treated with TAS-102 plus bevacizumab versus TAS-102 alone. A total of 44.1% of the patients included in the study were ≥ 65 years of age, and the subgroup analysis favoured the combination in this subgroup of patients.

Regarding treatment in patients with *BRAF* V600E-mutated tumours, the phase III BEACON trial with patients with *BRAF*-mutated CRC randomized patients who had progressed on first- and second-line treatments to receive encorafenib, an oral inhibitor of *BRAF* V600E, with cetuximab with or without binimetinib versus CT (FOLFIRI or irinotecan) plus cetuximab. The experimental arms exhibited benefits in OS compared to the control arm, establishing the encorafenib-cetuximab combination as the preferred regimen for these patients. In this study, 34.8% of the patients were ≥ 65 years of age (age range: 30–91 years in the encorafenib-cetuximab arm), and the subgroup analysis indicated a benefit in OS for patients ≥ 65 years of age in the experimental branch [75].

In patients with human epidermal growth factor receptor 2 (*HER2*) overexpression, i.e., between 3 and 5% of CRC patients, anti-*HER2* treatments (trastuzumab plus lapatinib and trastuzumab plus pertuzumab) can be used, such as tucatinib or trastuzumab-deruxtecan.

Immunotherapy has shown greater efficacy than CT in patients with metastatic CRC with microsatellite instability or DNA repair deficiencies (dMMR). The phase II KEYNOTE 164 study evaluated efficacy and safety in two cohorts (cohort A with two previous lines and cohort B with one previous line). Twenty-eight percent of the patients were over 65 years of age in cohort A and 38% in cohort B; the response rate, which was the main outcome of the study, was 33% in both cohorts; and there were 13% and 16% grade 3 adverse events in each cohort, respectively [82]. Nivolumab, an anti-programmed cell death protein 1 (anti-PD-1) antibody, both in monotherapy and in combination with ipilimumab (cytotoxic T-lymphocyte antigen 4, anti-CTLA-4), demonstrated efficacy in pretreated patients. In both studies, more than 30% of the patients were older than 65 years of age. The percentage of treatment-related grade 3 adverse events was higher with the combination treatment [83, 84].

### Radical treatment of oligometastatic disease

Local ablative treatments (LAT) as well as locoregional therapies offer local tumour control and extensive cytoreduction with low morbidity and mortality. In oligometastatic disease, LAT can achieve long-term disease control by complete tumour ablation in patients not eligible for surgery [85]. Locoregional therapies such as Y90 radioembolization (RE) may contribute to the OS of selected patients by improving the local response in liver-dominant disease or by providing a salvage treatment in chemo-refractory disease [86]. These aspects have been included in the main international clinical guidelines for CRC with oligometastatic disease or liver dominant chemo-refractory metastases [87, 88], but in the context of elderly patients, efficacy data are still rare.

Yang S. et al*.* built a Markiv-decision model to examine the effect on life expectancy and quality concluding that, in older patients with comorbidities, RT may provide better results [89]. Another study reported by Tanis E. et al*.* in the phase II Clocc trial [90] included 119 patients with non-resectable colorectal liver metastases randomized to systemic treatment or systemic treatment plus radiofrequency, which met the primary end point of 30 months OS > 38%, with the absence of a subanalysis in older patients.

Chemoembolization (CE) is another LAT, including hepatic transarterial CE (TACE) therapy with drug-eluting beads loaded with irinotecan (DEBIRI), that has been used in several prospective studies that demonstrated improved OS (22 vs. 15 months; *p* = 0.031) with an acceptable toxicity profile [91]. Other studies have shown benefits with the simultaneous administration of CT and DEBIRI in response rate (78% vs. 54% at 2 months; *p* = 0.02) [92].

Considering locoregional therapies such as radioembolization, Seidenstickler et al*.* reported the results of a cohort study including 266 patients who received radiofrequency, high-dose rate brachytherapy (HDR-BT) or Y90-RE. Median OS was 14 months and age > 70 years did not influence survival after local therapies [93].

Finally, SBRT offers promising results. Recently, the SABR-COMET study showed a median OS of 3.2 months compared with 1.6 months in the control group of elderly patients treated with SBRT with a response rate per adverse events grade ≥ 2 of 22.2% compared with 21.4% in the control group [94]. There are no results of phase III trials at this point, but several phase II studies are ongoing, and results are expected in the near future.

In conclusion, LAT can be selected for elderly CRC patients with liver and lung metastases who are not suitable for surgery. CE and RE are reasonable options in selected elderly patients with CRC and predominantly hepatic metastasis.

## Geriatric interventions

After performing a CGA, an intervention plan must be developed considering compromised or vulnerable domains that have been detected. Several domains are considered very important from the oncological point of view (Fig. 3). One domain is nutrition, and the objective of nutrition is to avoid malnutrition syndrome, sarcopenia and cachexia. For this, there are 3 possible interventions, which are not mutually exclusive: (i) individualized nutritional advice; (ii) nutritional support with oral supplements; and (iii) pharmaconutrients. To establish the composition of the supplements and their content in pharmaconutrients, the catabolic-inflammatory situation of patients should be taken into account [95, 96].

The functionality and independence of a patient improves with physical exercise [97]. The Vivifrail team provides a list of exercises to be performed based on the functional situation of each patient [98]. Different studies have shown that the combination of multimodal exercises with nutritional support leads to better results in the recovery of lean mass and in functionality; therefore, both are recommended to be provided together.

Prehabilitation is another domain that involves a multimodal process and that allows functional recovery in stressful situations. Patients should not be excluded from subsequent rehabilitation [99]. In most centres, in digestive pathology, and therefore in colon pathology, enhanced recovery after surgery (ERAS) care protocols are followed as presurgical prehabilitation programmes [100]. In addition, the treatment of chronic diseases before starting cancer treatment should be optimized [14].

Patients usually have complex therapeutic requirements, with visits to multiple specialists and for tests, for which they need optimal social and family support. It must be taken into account that in many cases, patients may have only one main caregiver who may also be elderly. Other important domains in which it is necessary to intervene are polypharmacy, adapting to treatment, the management of depression-anxiety, and reducing the risk of cognitive impairment or delirium, which also play important roles from an oncological point of view.

## Future research

There are various ongoing clinical trials and projects, either active or recently completed, related to the systemic treatment of older patients with CRC.

In the adjuvant setting, the ADAGE-PRODIGE randomized phase III trial is comparing, for patients ≥ 70 years of age and in terms of PFS at 3 years, treatment with fluoropyrimidines and oxaliplatin versus only fluoropyrimidines in physically fit patients (group 1) and administration of fluoropyrimidines versus only observation in frail patients (group 2). Thus far, only preliminary tolerance data have been published for 50% of the patients included, showing that grade 3–5 toxicities were more common in fit individuals treated with oxaliplatin or in frail patients treated with fluoropyrimidines than in fit patients treated with fluoropyrimidines, and that early discontinuations were more frequent among frail patients [101].

For advanced disease, a multicentre controlled phase II clinical trial (NCT03530267), in which 196 elderly or frail patients with metastatic CRC have been included, is evaluating the administration of aflibercept with 5FU or FOLFOX (1:1) as the first line of treatment with an initial dose reduced to 80%. The main outcome is the rate of progression-free survival at 6 months.

In the phase II clinical trial CAIRO7 (NCT05092880), expected completion date October 2028, elderly or frail patients with CRC and unresectable liver metastases are randomized to receive a liver ER with holmium-166 microspheres as a first-line treatment or combined capecitabine and anti-*VEGF* treatment. The main outcome is PFS.

The SOLSTICE clinical trial (NCT03869892), already mentioned, includes patients with metastatic CRC who are not candidates for or who do not require intensive treatment.

All these investigations will help to improve the therapeutic options available for elderly or frail patients with CRC.

## Conclusions

CRC is the most common cancer in Europe, with 70% of patients older than 65 years of age. A multidisciplinary approach and performing a GA before each therapeutic decision is essential. GA predicts the risk of toxicity and survival and is of great help to tailor individualized treatment. In addition, it allows detection of patient vulnerabilities, allowing interventions aimed at achieving less morbidity and toxicity of the treatments administered. Elderly patients with localized CRC should undergo standard cancer resection, preferably laparoscopically.

There are few studies aimed at this population; therefore, the therapeutic recommendations are based on the extrapolation of the results obtained for the general population. The indication for adjuvant CT should be considered in the context of potential benefits, risk of recurrence and life expectancy in relation to age and comorbidities, as well as patient preferences. In fit elderly patients with stage III CRC, adjuvant CT with fluoropyrimidines can be administered for 6 months. In prefrail patients, capecitabine can be administered at reduced doses, and the patient should be closely monitored for toxicity.

When disease is metastatic, radical treatment with surgery or radiofrequency (RF) or SBRT should be considered. Regarding palliative CT, if a patient is fit, it is recommended that the same treatment as that for younger patients be used, and dose reductions assessed to minimize the incidence of toxicity while maintaining similar efficacy. In prefrail patients, if they do not have *RAS* and *BRAF* gene mutations, the combination of fluoropyrimidines with bevacizumab or anti-EGFR is a good option. In contrast, frail patients are candidates for symptomatic palliative treatment without CT.

In the future, specific clinical trials in the elderly population that include a GA and specific objectives for this population should be conducted. The Oncogeriatrics Section of the SEOM as well as other scientific associations are working on the dissemination and implementation of projects to strengthen the evidence and improve the assessment of elderly patients with cancer.

## Data Availability

Not applicable.
