# Correction: Update on the management of elderly patients with colorectal cancer

**DOI:** 10.1007/s12094-023-03351-x

**Published:** 2023-11-29

**Authors:** Gemma Soler-González, Javier Sastre-Valera, Antonio Viana-Alonso, Jorge Aparicio-Urtasun, Ignacio García-Escobar, María Auxiliadora Gómez-España, Carmen Guillén-Ponce, María José Molina-Garrido, Regina Gironés-Sarrió

**Affiliations:** 1 Departamento de Oncología Médica, Spanish Society of Medical Oncology (SEOM) Oncogeriatrics Section, Institut Català d’Oncologia (ICO) L’Hospitalet, Avinguda de la Granvia de l’Hospitalet 199-203, L’Hospitalet de Llobregat, 08908 Barcelona, Spain; 2 Spanish Cooperative Group for the Treatment of Digestive Tumours (TTD), Clinico San Carlos University Hospital, Madrid, Spain; 3 Spanish Society of Medical Oncology (SEOM) Oncogeriatrics Section, Nuestra Señora del Prado General University Hospital, Talavera de la Reina, Spain; 4 Multidisciplinary Spanish Group of Digestive Cancer (GEMCAD), Polytechnic la Fe University Hospital, Valencia, Spain; 5 Spanish Society of Medical Oncology (SEOM) Oncogeriatrics Section, General University Hospital of Toledo, Toledo, Spain; 6 Spanish Society of Medical Oncology (SEOM) Oncogeriatrics Section, Reina Sofía University Hospital. Instituto Maimónides de Investigación Biomédica de Córdoba (IMIBIC), Córdoba, Spain; 7 Spanish Society of Medical Oncology (SEOM) Oncogeriatrics Section, Ramón y Cajal University Hospital, Madrid, Spain; 8 Spanish Society of Medical Oncology (SEOM) Oncogeriatrics Section, Virgen de la Luz Hospital, Cuenca, Spain; 9 Spanish Society of Medical Oncology (SEOM) Oncogeriatrics Section, Polytechnic la Fe University Hospital, Valencia, Spain

**Correction to: Clinical and Translational Oncology** 10.1007/s12094-023-03243-0

In this article the funder name in the Funding information section was incorrectly given as ‘Merck’ and should have read ‘Merck, S.L.U., Spain, an affiliate of Merck KGaA, Darmstadt, Germany’.

The original article has been corrected.

